# Dichlorido[2,3,5,6-tetra­fluoro-4-(tri­fluoro­meth­yl)phenyl-κ*C* ^1^]bis­(tri­methyl­phosphine-κ*P*)cobalt(III)

**DOI:** 10.1107/S1600536810014066

**Published:** 2010-04-24

**Authors:** Tingting Zheng, Hongjian Sun

**Affiliations:** a School of Chemistry and Chemical Engineering, Shandong University, Jinan 250100, People’s Republic of China

## Abstract

In the title compound, [Co(C_7_F_7_)Cl_2_(C_3_H_9_P)_2_], the Co^III^ atom displays a trigonal–bipyramidal coordination geometry, with the two Cl ligands and the C atom of the perfluoro­tolyl ligand in the equatorial plane and the two phosphine ligands occupying axial positions. The mol­ecule has an approximate *C*_2v_ symmetry. The trifluoro­methyl group is disordered over two positions, with nearly equal occupancies.

## Related literature

For general background on the activation of C—F bonds and the formation of C—C bonds, see: Schaub *et al.* (2006 ▶); Böhm *et al.* (2001 ▶); Zheng *et al.* (2009 ▶).
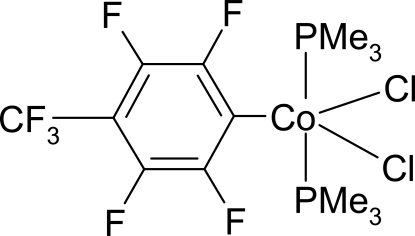


## Experimental

### 

#### Crystal data

[Co(C_7_F_7_)Cl_2_(C_3_H_9_P)_2_]
*M* *_r_* = 499.04
Orthorhombic, 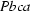

*a* = 12.3321 (19) Å
*b* = 13.3657 (19) Å
*c* = 25.426 (4) Å
*V* = 4190.8 (11) Å^3^
*Z* = 8
Mo *K*α radiation
μ = 1.28 mm^−1^
*T* = 293 K
0.25 × 0.20 × 0.18 mm

#### Data collection

Bruker SMART CCD area-detector diffractometer
Absorption correction: multi-scan (*SADABS*; Sheldrick, 1996 ▶) *T*_min_ = 0.740, *T*_max_ = 0.802
18353 measured reflections
3373 independent reflections
2345 reflections with *I* > 2σ(*I*)
*R*_int_ = 0.052

#### Refinement

*R*[*F* ^2^ > 2σ(*F* ^2^)] = 0.061
*wR*(*F* ^2^) = 0.186
*S* = 1.07
3373 reflections
223 parameters
8 restraints
H-atom parameters constrained
Δρ_max_ = 0.78 e Å^−3^
Δρ_min_ = −0.69 e Å^−3^

### 

Data collection: *SMART* (Bruker, 1997 ▶); cell refinement: *SAINT* (Bruker, 1997 ▶); data reduction: *SAINT*; program(s) used to solve structure: *SHELXS97* (Sheldrick, 2008 ▶); program(s) used to refine structure: *SHELXL97* (Sheldrick, 2008 ▶); molecular graphics: *SHELXTL* (Sheldrick, 2008 ▶); software used to prepare material for publication: *SHELXTL*.

## Supplementary Material

Crystal structure: contains datablocks I, global. DOI: 10.1107/S1600536810014066/gk2264sup1.cif

Structure factors: contains datablocks I. DOI: 10.1107/S1600536810014066/gk2264Isup2.hkl

Additional supplementary materials:  crystallographic information; 3D view; checkCIF report

## Figures and Tables

**Table:** 

C1—Co1	1.987 (6)
Cl1—Co1	2.2290 (18)
Cl2—Co1	2.2613 (16)
Co1—P1	2.262 (2)
Co1—P2	2.264 (2)

**Table:** 

C1—Co1—Cl1	123.2 (2)
C1—Co1—Cl2	125.5 (2)
Cl1—Co1—Cl2	111.29 (7)
C1—Co1—P1	89.36 (19)
Cl1—Co1—P1	90.78 (8)
Cl2—Co1—P1	90.03 (7)
C1—Co1—P2	89.93 (19)
Cl1—Co1—P2	89.13 (8)
Cl2—Co1—P2	90.86 (7)
P1—Co1—P2	179.08 (8)

**Fig. 1.**
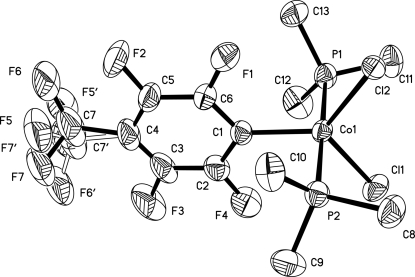
Molecular structure and aAtom numbering scheme for the title compound with the displacement ellipsoids shown at the 30% probability level.

**Fig. 2.**
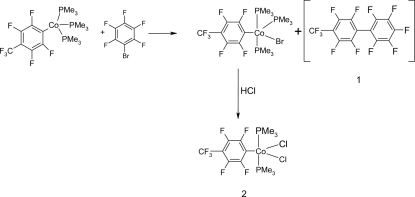
Preparation of the title compound.

## supplementary crystallographic information

### Comment

The activation of C—F bonds by transition metal compounds has blossomed in the past few years. There has also been considerable interest in the chemistry of carbon-fluorine bond cleavage followed by carbon-carbon bond formation because they play a key role in the organic synthesis (Schaub *et al.*, 2006; Böhm *et al.*, 2001). Recently we have reported the stoichiometric reaction involving one-electron oxidative addition of bromobenzene and (1-perflurotoluene-κ*C*)tris(trimethylphosphine-κ*P*)cobalt gaining bromo(1-perflurotoluene-κ*C*)tris(trimethylphosphine-κ*P*)cobalt and C—C coupling product (Zheng *et al.*, 2009). We tried to synthesise the compound 1, through the reaction of bromopentafluorobenzene with the(1-perflurotoluene-κ*C*)tris(trimethylphosphine-κ*P*)cobalt. We added the solution of hydrochloric acid in order to abolish the organometallic compounds and gain the organic compound 1. Surprisingly we isolated complex 2 ( Scheme 2) as red crystals and its molecular structure is shown in Fig.1. The cobalt atom displays a trigonal bipyramidal coordination, with two Cl atoms and C atom in the equatorial plane and two P atoms occupying axial positions. The Cl1—Co1 and Cl2—Co1) distances are 2.2290 (18) Å and 2.2613 (16) Å, respectively. The angle between the phosphine ligands and the Co atom, P1–Co1–P2 is 179.08 (8)°.

### Experimental

The reaction leading to the title compound is shown in Scheme 2. To a solution of 1 (0.50 g,1.00 mmol) in 30 mL of pentane was added bromopentafluorobenzene (0.74 g, 3.00 mmol) with stirring at 213 K. The mixture was allowed to warm-up to 293 K and was stirred for 18 h. The color changed from green to yellow-brown. The reaction mixture was added to a solution of hydrochloric acid with a color change to red-brown. Pentane was used to estract the organic product. Crystallization from pentane at 273 K afforded the title compound as red crystals in 37% yield.

### Refinement

Hydrogen atoms were included in the refinement at calculated positions (C–H = 0.97 Å) and treated as riding, with *U*_iso_(H) =1.5 *U*_eq_(C). In the refinement process, the sum of the occupancy  factors of the disordered CF_3_ groups was constrained to 1.0 and restrains were imposed on its geometry [C-C 1.54 (2) Å;  C-F 1.36 (2) Å]. The occupancy factor of the major orientation of the CF_3  _group refined at 0.513 (13).

### Crystal data

**Table:**

[Co(C_7_F_7_)Cl_2_(C_3_H_9_P)_2_]	*D*_x_ = 1.582 Mg m^−^^3^
*M**_r_* = 499.04	Melting point: 380 K
Orthorhombic, *P**b**c**a*	Mo *K*α radiation, λ = 0.71073 Å
Hall symbol: -P 2ac 2ab	Cell parameters from 2967 reflections
*a* = 12.3321 (19) Å	θ = 2.3–21.0°
*b* = 13.3657 (19) Å	µ = 1.28 mm^−^^1^
*c* = 25.426 (4) Å	*T* = 293 K
*V* = 4190.8 (11) Å^3^	Block, red
*Z* = 8	0.25 × 0.20 × 0.18 mm
*F*(000) = 2000	

### Data collection

**Table:**

Bruker SMART CCD area-detector diffractometer	3373 independent reflections
Radiation source: fine-focus sealed tube	2345 reflections with *I* > 2σ(*I*)
graphite	*R*_int_ = 0.052
φ and ω scans	θ_max_ = 24.2°, θ_min_ = 2.3°
Absorption correction: multi-scan (*SADABS*; Sheldrick, 1996)	*h* = −14→10
*T*_min_ = 0.740, *T*_max_ = 0.802	*k* = −15→12
18353 measured reflections	*l* = −27→29

### Refinement

**Table:**

Refinement on *F*^2^	Primary atom site location: structure-invariant direct methods
Least-squares matrix: full	Secondary atom site location: difference Fourier map
*R*[*F*^2^ > 2σ(*F*^2^)] = 0.061	Hydrogen site location: inferred from neighbouring sites
*wR*(*F*^2^) = 0.186	H-atom parameters constrained
*S* = 1.07	*w* = 1/[σ^2^(*F*_o_^2^) + (0.0935*P*)^2^ + 7.5678*P*] where *P* = (*F*_o_^2^ + 2*F*_c_^2^)/3
3373 reflections	(Δ/σ)_max_ = 0.013
223 parameters	Δρ_max_ = 0.78 e Å^−^^3^
8 restraints	Δρ_min_ = −0.69 e Å^−^^3^

### Special details

**Table:**

Geometry. All esds (except the esd in the dihedral angle between two l.s. planes) are estimated using the full covariance matrix. The cell esds are taken into account individually in the estimation of esds in distances, angles and torsion angles; correlations between esds in cell parameters are only used when they are defined by crystal symmetry. An approximate (isotropic) treatment of cell esds is used for estimating esds involving l.s. planes.
Refinement. Refinement of *F*^2^ against ALL reflections. The weighted *R*-factor wR and goodness of fit *S* are based on *F*^2^, conventional *R*-factors *R* are based on *F*, with *F* set to zero for negative *F*^2^. The threshold expression of *F*^2^ > σ(*F*^2^) is used only for calculating *R*-factors(gt) etc. and is not relevant to the choice of reflections for refinement. *R*-factors based on *F*^2^ are statistically about twice as large as those based on *F*, and *R*- factors based on ALL data will be even larger.

### Fractional atomic coordinates and isotropic or equivalent isotropic displacement parameters (Å^2^)

**Table:**

	*x*	*y*	*z*	*U*_iso_*/*U*_eq_	Occ. (<1)
C1	0.9572 (5)	0.1013 (5)	0.1322 (3)	0.0593 (16)	
C2	0.9141 (7)	0.1728 (6)	0.0990 (3)	0.080 (2)	
C3	0.8099 (8)	0.1731 (9)	0.0813 (3)	0.102 (3)	
C4	0.7378 (6)	0.0992 (11)	0.0944 (4)	0.109 (4)	
C5	0.7789 (7)	0.0239 (8)	0.1266 (3)	0.094 (3)	
C6	0.8834 (6)	0.0266 (6)	0.1449 (3)	0.073 (2)	
C8	1.3056 (6)	−0.0189 (6)	0.0919 (3)	0.087 (2)	
H8A	1.3203	−0.0538	0.1241	0.130*	
H8B	1.3477	0.0414	0.0907	0.130*	
H8C	1.3244	−0.0607	0.0626	0.130*	
C9	1.1447 (8)	0.0770 (7)	0.0268 (3)	0.101 (3)	
H9A	1.1794	0.1413	0.0286	0.151*	
H9B	1.0687	0.0858	0.0201	0.151*	
H9C	1.1766	0.0384	−0.0010	0.151*	
C10	1.0943 (7)	−0.1069 (5)	0.0800 (3)	0.087 (2)	
H10A	1.1015	−0.1461	0.1115	0.130*	
H10B	1.1264	−0.1422	0.0510	0.130*	
H10C	1.0189	−0.0954	0.0729	0.130*	
C11	1.1647 (6)	0.2311 (6)	0.2713 (3)	0.092 (3)	
H11A	1.2031	0.1723	0.2824	0.137*	
H11B	1.1369	0.2656	0.3016	0.137*	
H11C	1.2131	0.2744	0.2524	0.137*	
C12	0.9874 (8)	0.3117 (6)	0.2110 (4)	0.105 (3)	
H12A	1.0344	0.3495	0.1884	0.157*	
H12B	0.9727	0.3495	0.2423	0.157*	
H12C	0.9206	0.2979	0.1931	0.157*	
C13	0.9579 (7)	0.1302 (8)	0.2711 (3)	0.101 (3)	
H13A	0.9885	0.0672	0.2816	0.151*	
H13B	0.8913	0.1187	0.2525	0.151*	
H13C	0.9438	0.1701	0.3017	0.151*	
Cl1	1.22875 (15)	0.22034 (13)	0.13739 (8)	0.0772 (6)	
Cl2	1.18147 (13)	−0.01081 (11)	0.21464 (6)	0.0566 (4)	
Co1	1.10845 (6)	0.10295 (6)	0.15914 (3)	0.0498 (3)	
F1	0.9169 (4)	−0.0490 (4)	0.17618 (19)	0.0965 (15)	
F2	0.7167 (4)	−0.0546 (6)	0.1418 (2)	0.146 (3)	
F3	0.7792 (5)	0.2497 (5)	0.0496 (2)	0.156 (3)	
F4	0.9791 (5)	0.2502 (4)	0.0832 (2)	0.1083 (16)	
P1	1.05246 (15)	0.19513 (14)	0.22862 (7)	0.0664 (5)	
P2	1.16269 (14)	0.01209 (14)	0.08875 (7)	0.0622 (5)	
C7	0.6149 (13)	0.0863 (14)	0.0797 (8)	0.106 (9)*	0.487 (13)
F6	0.5612 (14)	0.0191 (12)	0.1062 (6)	0.154 (7)*	0.487 (13)
F5	0.5737 (12)	0.1839 (12)	0.0899 (6)	0.138 (6)*	0.487 (13)
F7	0.6077 (13)	0.0920 (13)	0.0284 (6)	0.150 (6)*	0.487 (13)
C7'	0.6232 (14)	0.1080 (15)	0.0695 (8)	0.141 (12)*	0.513 (13)
F6'	0.6148 (12)	0.1727 (13)	0.0306 (6)	0.158 (6)*	0.513 (13)
F7'	0.6005 (11)	0.0140 (10)	0.0511 (5)	0.133 (5)*	0.513 (13)
F5'	0.5527 (11)	0.1180 (11)	0.1069 (5)	0.131 (5)*	0.513 (13)

### Atomic displacement parameters (Å^2^)

**Table:**

	*U*^11^	*U*^22^	*U*^33^	*U*^12^	*U*^13^	*U*^23^
C1	0.046 (4)	0.069 (4)	0.062 (4)	0.006 (3)	−0.001 (3)	−0.001 (3)
C2	0.072 (5)	0.092 (6)	0.077 (5)	0.023 (5)	−0.004 (4)	0.012 (4)
C3	0.078 (6)	0.152 (9)	0.074 (5)	0.056 (7)	−0.024 (5)	−0.011 (6)
C4	0.042 (4)	0.209 (12)	0.076 (6)	0.027 (6)	−0.010 (4)	−0.035 (7)
C5	0.052 (5)	0.157 (9)	0.072 (5)	−0.024 (6)	0.013 (4)	−0.030 (6)
C6	0.053 (4)	0.095 (6)	0.072 (5)	−0.008 (4)	0.003 (4)	0.004 (4)
C8	0.055 (4)	0.104 (6)	0.101 (6)	0.014 (4)	0.009 (4)	−0.010 (5)
C9	0.121 (7)	0.113 (7)	0.068 (5)	0.018 (6)	0.002 (5)	0.006 (5)
C10	0.083 (6)	0.072 (5)	0.105 (6)	0.012 (4)	−0.018 (5)	−0.019 (4)
C11	0.075 (5)	0.096 (6)	0.103 (6)	0.012 (5)	−0.017 (5)	−0.031 (5)
C12	0.111 (7)	0.081 (6)	0.122 (7)	0.042 (5)	−0.003 (6)	−0.018 (5)
C13	0.078 (6)	0.148 (8)	0.078 (5)	−0.005 (6)	0.021 (4)	−0.005 (5)
Cl1	0.0681 (11)	0.0602 (10)	0.1034 (14)	−0.0203 (9)	0.0083 (10)	0.0143 (9)
Cl2	0.0546 (9)	0.0522 (9)	0.0629 (9)	0.0059 (7)	−0.0052 (7)	0.0159 (7)
Co1	0.0414 (5)	0.0477 (5)	0.0603 (5)	0.0008 (3)	0.0002 (4)	0.0075 (4)
F1	0.088 (3)	0.098 (3)	0.103 (3)	−0.031 (3)	−0.006 (3)	0.027 (3)
F2	0.076 (3)	0.227 (7)	0.136 (5)	−0.073 (5)	0.010 (3)	−0.025 (5)
F3	0.138 (5)	0.199 (6)	0.130 (5)	0.083 (5)	−0.048 (4)	0.023 (4)
F4	0.112 (4)	0.089 (3)	0.124 (4)	0.017 (3)	−0.012 (3)	0.042 (3)
P1	0.0549 (11)	0.0721 (12)	0.0723 (11)	0.0116 (9)	0.0003 (9)	−0.0081 (9)
P2	0.0544 (10)	0.0683 (11)	0.0638 (11)	0.0091 (9)	−0.0010 (8)	0.0011 (8)

### Geometric parameters (Å, °)

**Table:**

C1—C2	1.382 (10)	C10—H10C	0.9600
C1—C6	1.389 (10)	C11—P1	1.823 (8)
C1—Co1	1.987 (6)	C11—H11A	0.9600
C2—C3	1.362 (12)	C11—H11B	0.9600
C2—F4	1.369 (9)	C11—H11C	0.9600
C3—F3	1.358 (11)	C12—P1	1.808 (8)
C3—C4	1.370 (15)	C12—H12A	0.9600
C4—C5	1.393 (14)	C12—H12B	0.9600
C4—C7'	1.554 (17)	C12—H12C	0.9600
C4—C7	1.570 (16)	C13—P1	1.811 (8)
C5—F2	1.357 (11)	C13—H13A	0.9600
C5—C6	1.370 (11)	C13—H13B	0.9600
C6—F1	1.350 (9)	C13—H13C	0.9600
C8—P2	1.812 (7)	Cl1—Co1	2.2290 (18)
C8—H8A	0.9600	Cl2—Co1	2.2613 (16)
C8—H8B	0.9600	Co1—P1	2.262 (2)
C8—H8C	0.9600	Co1—P2	2.264 (2)
C9—P2	1.812 (8)	C7—F6	1.302 (16)
C9—H9A	0.9600	C7—F7	1.311 (17)
C9—H9B	0.9600	C7—F5	1.424 (17)
C9—H9C	0.9600	C7'—F5'	1.296 (17)
C10—P2	1.814 (8)	C7'—F6'	1.317 (17)
C10—H10A	0.9600	C7'—F7'	1.368 (17)
C10—H10B	0.9600		
			
C2—C1—C6	112.8 (7)	P1—C12—H12B	109.5
C2—C1—Co1	124.3 (6)	H12A—C12—H12B	109.5
C6—C1—Co1	122.9 (5)	P1—C12—H12C	109.5
C3—C2—F4	117.0 (8)	H12A—C12—H12C	109.5
C3—C2—C1	124.5 (9)	H12B—C12—H12C	109.5
F4—C2—C1	118.5 (7)	P1—C13—H13A	109.5
F3—C3—C2	117.5 (11)	P1—C13—H13B	109.5
F3—C3—C4	120.4 (9)	H13A—C13—H13B	109.5
C2—C3—C4	122.1 (9)	P1—C13—H13C	109.5
C3—C4—C5	115.3 (7)	H13A—C13—H13C	109.5
C3—C4—C7'	115.9 (12)	H13B—C13—H13C	109.5
C5—C4—C7'	128.7 (13)	C1—Co1—Cl1	123.2 (2)
C3—C4—C7	130.4 (12)	C1—Co1—Cl2	125.5 (2)
C5—C4—C7	114.3 (12)	Cl1—Co1—Cl2	111.29 (7)
C7'—C4—C7	14.8 (11)	C1—Co1—P1	89.36 (19)
F2—C5—C6	117.1 (9)	Cl1—Co1—P1	90.78 (8)
F2—C5—C4	121.3 (8)	Cl2—Co1—P1	90.03 (7)
C6—C5—C4	121.6 (9)	C1—Co1—P2	89.93 (19)
F1—C6—C5	118.0 (8)	Cl1—Co1—P2	89.13 (8)
F1—C6—C1	118.3 (6)	Cl2—Co1—P2	90.86 (7)
C5—C6—C1	123.8 (8)	P1—Co1—P2	179.08 (8)
P2—C8—H8A	109.5	C12—P1—C13	105.9 (5)
P2—C8—H8B	109.5	C12—P1—C11	104.9 (4)
H8A—C8—H8B	109.5	C13—P1—C11	105.1 (4)
P2—C8—H8C	109.5	C12—P1—Co1	114.3 (3)
H8A—C8—H8C	109.5	C13—P1—Co1	113.6 (3)
H8B—C8—H8C	109.5	C11—P1—Co1	112.1 (3)
P2—C9—H9A	109.5	C9—P2—C8	105.5 (4)
P2—C9—H9B	109.5	C9—P2—C10	104.9 (4)
H9A—C9—H9B	109.5	C8—P2—C10	104.9 (4)
P2—C9—H9C	109.5	C9—P2—Co1	113.2 (3)
H9A—C9—H9C	109.5	C8—P2—Co1	112.0 (3)
H9B—C9—H9C	109.5	C10—P2—Co1	115.5 (3)
P2—C10—H10A	109.5	F6—C7—F7	121.3 (17)
P2—C10—H10B	109.5	F6—C7—F5	110.9 (16)
H10A—C10—H10B	109.5	F7—C7—F5	96.0 (15)
P2—C10—H10C	109.5	F6—C7—C4	116.3 (16)
H10A—C10—H10C	109.5	F7—C7—C4	107.2 (14)
H10B—C10—H10C	109.5	F5—C7—C4	101.6 (13)
P1—C11—H11A	109.5	F5'—C7'—F6'	115.5 (17)
P1—C11—H11B	109.5	F5'—C7'—F7'	102.1 (16)
H11A—C11—H11B	109.5	F6'—C7'—F7'	109.3 (16)
P1—C11—H11C	109.5	F5'—C7'—C4	108.5 (15)
H11A—C11—H11C	109.5	F6'—C7'—C4	115.3 (16)
H11B—C11—H11C	109.5	F7'—C7'—C4	104.8 (14)
P1—C12—H12A	109.5		
			
C6—C1—C2—C3	1.7 (11)	C1—Co1—P1—C12	−57.7 (4)
Co1—C1—C2—C3	−178.6 (6)	Cl1—Co1—P1—C12	65.5 (4)
C6—C1—C2—F4	−179.5 (7)	Cl2—Co1—P1—C12	176.8 (4)
Co1—C1—C2—F4	0.2 (10)	C1—Co1—P1—C13	64.1 (4)
F4—C2—C3—F3	0.1 (12)	Cl1—Co1—P1—C13	−172.7 (3)
C1—C2—C3—F3	178.9 (7)	Cl2—Co1—P1—C13	−61.4 (3)
F4—C2—C3—C4	179.7 (8)	C1—Co1—P1—C11	−176.9 (4)
C1—C2—C3—C4	−1.5 (14)	Cl1—Co1—P1—C11	−53.7 (3)
F3—C3—C4—C5	179.1 (7)	Cl2—Co1—P1—C11	57.6 (3)
C2—C3—C4—C5	−0.4 (13)	C1—Co1—P2—C9	63.6 (4)
F3—C3—C4—C7'	1.8 (15)	Cl1—Co1—P2—C9	−59.6 (4)
C2—C3—C4—C7'	−177.8 (11)	Cl2—Co1—P2—C9	−170.8 (4)
F3—C3—C4—C7	−2.0 (17)	C1—Co1—P2—C8	−177.3 (4)
C2—C3—C4—C7	178.5 (12)	Cl1—Co1—P2—C8	59.6 (3)
C3—C4—C5—F2	−178.9 (8)	Cl2—Co1—P2—C8	−51.7 (3)
C7'—C4—C5—F2	−1.9 (16)	C1—Co1—P2—C10	−57.3 (3)
C7—C4—C5—F2	2.0 (14)	Cl1—Co1—P2—C10	179.5 (3)
C3—C4—C5—C6	2.0 (13)	Cl2—Co1—P2—C10	68.2 (3)
C7'—C4—C5—C6	179.0 (12)	C3—C4—C7—F6	−166.9 (13)
C7—C4—C5—C6	−177.1 (10)	C5—C4—C7—F6	12.0 (19)
F2—C5—C6—F1	0.5 (11)	C7'—C4—C7—F6	180 (5)
C4—C5—C6—F1	179.6 (7)	C3—C4—C7—F7	54 (2)
F2—C5—C6—C1	179.0 (7)	C5—C4—C7—F7	−127.5 (14)
C4—C5—C6—C1	−1.8 (13)	C7'—C4—C7—F7	40 (4)
C2—C1—C6—F1	178.4 (7)	C3—C4—C7—F5	−46.5 (19)
Co1—C1—C6—F1	−1.3 (10)	C5—C4—C7—F5	132.4 (12)
C2—C1—C6—C5	−0.1 (11)	C7'—C4—C7—F5	−60 (4)
Co1—C1—C6—C5	−179.8 (6)	C3—C4—C7'—F5'	−118.0 (15)
C2—C1—Co1—Cl1	−1.3 (7)	C5—C4—C7'—F5'	65 (2)
C6—C1—Co1—Cl1	178.4 (5)	C7—C4—C7'—F5'	51 (4)
C2—C1—Co1—Cl2	178.8 (5)	C3—C4—C7'—F6'	13 (2)
C6—C1—Co1—Cl2	−1.5 (7)	C5—C4—C7'—F6'	−163.6 (13)
C2—C1—Co1—P1	89.2 (6)	C7—C4—C7'—F6'	−178 (5)
C6—C1—Co1—P1	−91.1 (6)	C3—C4—C7'—F7'	133.6 (13)
C2—C1—Co1—P2	−90.2 (6)	C5—C4—C7'—F7'	−43.3 (19)
C6—C1—Co1—P2	89.5 (6)	C7—C4—C7'—F7'	−58 (4)
